# Thyrotropin N-glycosylation and Glycan Composition in Severe Primary Hypothyroidism

**DOI:** 10.1210/jendso/bvab006

**Published:** 2021-02-04

**Authors:** Leif Wide, Karin Eriksson

**Affiliations:** Department of Medical Sciences, Uppsala University, Clinical Chemistry, University Hospital, Uppsala, Sweden

**Keywords:** primary hypothyroidism, N-glycosylation, TSH glycoforms, sulfonated N- acetylgalactosamine, sialic acid, levothyroxine

## Abstract

**Context:**

In severe primary hypothyroidism (sPH), the serum thyrotropin (TSH) levels are elevated with an increased degree of sialylation. The circulating TSH comprises 2 different TSH glycoforms: TSHdi with 2 and TSHtri with 3 N-glycans and methods have developed to determine their contents of anionic monosaccharides (AMS), that is, sialic acid (SA) and sulfonated N-acetylglactosamine (SU) residues.

**Objective:**

Characterize N-glycosylation and glycan composition of circulating TSH molecules and determine the effects during levothyroxine treatment in patients with sPH.

**Methods:**

Serum samples were obtained from 25 patients with sPH, from 159 euthyroid individuals, and from 12 women during treatment with levothyroxine for sPH. Degrees of N-glycosylation and concentrations of TSHdi and TSHtri as well as their contents of AMS, SA, and SU residues were determined.

**Results:**

The circulating TSH molecules in sPH patients had lower degrees of N-glycosylation, higher degrees of sialylation, and lower degrees of sulfonation than in euthyroid individuals. Levothyroxin restored sialylation and sulfonation of the glycans already at low free thyroxine (FT4) levels, while degree of N-glycosylation was not restored until the FT4 levels were normal.

**Conclusions:**

The majority of TSH molecules in severe primary hypothyroidism were less N- glycosylated, more sialylated, and less sulfonated compared with euthyroid individuals. This glycan pattern favors a prolonged half-life in the circulation combined with lower in vitro biopotency at the target cells. During levothyroxine treatment of sPH patients, the sialylation and sulfonation of glycans were restored already at low FT4 levels, while N-glycosylation of TSH was not restored until the FT4 levels were normal.

Patients with severe primary hypothyroidism have an increased secretion of thyrotropin (thyroid-stimulating hormone; TSH) molecules from the pituitary and these molecules have been shown to be highly sialylated, contributing to a prolonged half-life and further raised TSH concentrations in the circulation [1-6]. The intrinsic bioactivity, as analyzed with in vitro bioassays, of these highly sialylated TSH molecules was decreased compared with TSH molecules of euthyroid individuals [7, 8].

TSH, like follicle-stimulating hormone (FSH) and luteinizing hormone (LH), is a pituitary glycoprotein hormone, which circulates as low- and fully N-glycosylated glycoforms [9]. The low-N-glycosylated form, TSHdi, has 2 N-glycans, while the fully N-glycosylated form, TSHtri, has 3 N-glycans. Both these TSH glycoforms exhibit a large heterogeneity due to variations in the decoration of the N-glycans with different number of 2 terminal anionic monosaccharides (AMS): sialic acid (SA) and sulfonated N-acetylgalactosamine (SU).

The N-glycosylation of TSH alpha- and beta-subunit polypeptides occurring co-translationally in the rough endoplasmic reticulum and the branching of the glycans and their decorations with terminal SA and SU residues in the Golgi of specific human anterior pituitary gland cells are schematically illustrated in Fig. 1; nomenclature, pathways and design from references [10-14].

**Figure 1.**
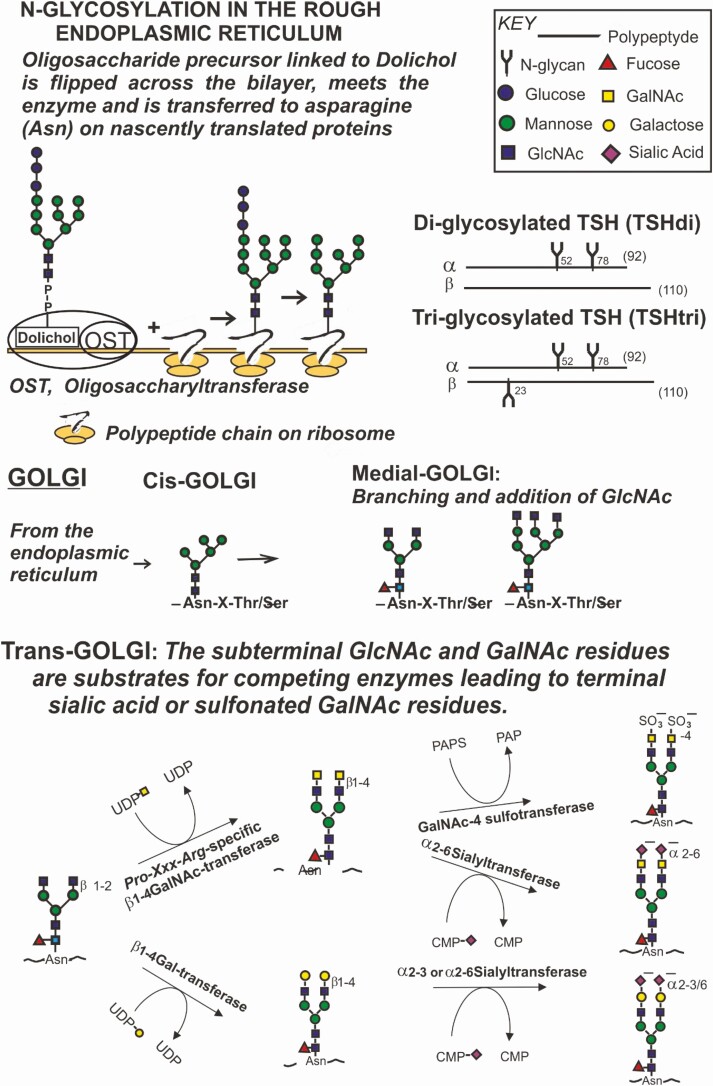
N-glycosylation of human TSH occurs in the rough endoplasmic reticulum (ER) of the thyrotrophs in the anterior pituitary gland. Within the Golgi apparatus in these cells, the glycans are then branched, followed by synthesis up to terminal sialic acid (SA) or sulfonated GalNAc (SU) residues. Oligosaccharide precursors linked to dolichol, a specific lipid, at the cytoplasmic side of the ER membrane are flipped across the membrane bilayer to the luminal side of the ER by use of an enzyme, a flippase. An enzymatic complex in the ER membrane, termed oligosaccharyltransferase (OST), transfers the oligosaccharide precursor to a gamma amino group of asparagine (-Asn-X-Thr/Ser) on the nascently translated TSH alpha- and beta-polypeptides. Fully N- glycosylated alpha-subunits, with 2 N-glycans, combine with the TSH beta-subunits with 1 or no N-glycan, forming TSHtri and TSHdi molecules, respectively. The TSH molecules are posttranslationally modified in the Golgi apparatus within the cell where the branching of the N-glycans occurs in the Medial-Golgi and their decoration with the 2 terminal anionic monosaccharide (AMS) residues: SA and SU in the Trans-Golgi. Abbreviations: CMP, cytidine 5’-monophosphate; PAPS, 3’phosphoadenyl-5’phosphosulfate; UDP, uridine diphosphate.

We have developed methods to identify the 2 circulating glycoforms of human TSH and characterized them concerning number of SA and SU residues per molecule [9]. Studies on euthyroid individuals, both children and adults, revealed that in children up to 18 months of age the circulating TSH molecules displayed a unique pattern of N-glycosylation, sialylation, and sulfonation [15]. There is hitherto no report on the frequencies of the 2 TSH glycoforms in serum in severe primary hypothyroidism and their numbers of SA and SU residues per molecule.

In the present study, the serum concentrations of TSHdi and TSHtri, the relative frequencies of the 2 glycoforms, and the numbers of SA, SU, and AMS residues per molecule were estimated in 25 patients with severe primary hypothyroidism. The results were compared with those of euthyroid individuals. The glycobiology of circulating TSH molecules was also determined during levothyroxine treatment in 12 women with severe primary hypothyroidism.

## Participants and Methods

### Participants

One serum sample was obtained from each of 25 patients with untreated severe primary hypothyroidism: 14 women aged 20 to 42 years; 5 women aged 51 to 74 years; and 6 men aged 59 to 93 years. The euthyroid control groups comprised 143 euthyroid female medical students, 21 to 44 years of age; 10 women, 46 to 80 years; and 6 men, 52 to 87 years. The samples were obtained during the period 1999 to 2012. The 25 patients with severe primary hypothyroidism had elevated TSH serum levels, between 42 and 544 mIU/L, and lowered free thyroxine (FT4) serum levels, between 1 and 3 pmol/L, when initially analyzed with the clinical chemical routine methods. The euthyroid individuals had both TSH and FT4 serum levels within the reference ranges of healthy individuals.

Hospital ward medical records for patients and health declarations from the medical students, made at the time the serum samples were taken, was studied by one of us (L.W.). The patients with severe primary hypothyroidism were untreated and none of the 159 euthyroid individuals had a known thyroid or pituitary disease.

One serum sample was also obtained from each of 12 women treated with levothyroxine for severe primary hypothyroidism. The group of women treated with levothyroxine was subdivided into 3 groups, each including 4 women, according to their FT4 serum concentrations, with mean values of 6.0 (LevoT4-A), 11.0 (LevoT4-B), and 17.2 (LevoT4-C) pmol/L, respectively. Reference groups of serum samples comprised age-matched subsets from the female groups of individuals described above: 16 from the groups of women with untreated severe primary hypothyroidism and 38 from the groups of euthyroid women. The mean values and ranges for age and for FT4 and TSH concentrations of these 3 groups and of the reference groups of 38 euthyroid women and of 16 women with untreated severe hypothyroidism are presented in Table 1.

**Table 1.** Age-Matched Groups of Women With Severe Primary Hypothyroidism, Nontreated and Treated with Levothyroxine, and of Euthyroid Women

Group	Age, mean (range), years	Number	Free thyroxine (FT4), mean (range), pmol/L	TSH, mean (range), IU/L
**Nontreated severe primary hypothyroidism**				
Group sPH	42.6 (27–74)	16	2.5 (1.0–3.0)	227 (42–544)
**Levothyroxine treatment**				
Group LevoT4-A	43.8 (33–57)	4	6.0 (4.1–7.4)	137 (48–267)
Group LevoT4-B	44.8 (29–84)	4	11.0 (10.5–11.8)	21 (7.1–32)
Group LevoT4-C	42.0 (27–74)	4	17.2 (14.2–22)	4.0 (0.6–9.9)
**Euthyroid individuals**				
Group Euthyroid	42.1 (30–80)	38	14.1 (12.0–22)	2.0 (1.0–4.6)

Abbreviations: LevoT4-A, group with FT4 serum concentrations mean value of 6.0 pmol/L; LevoT4-B, group with FT4 serum concentrations mean value of 11.0 pmol/L; LevoT4-C, group with FT4 serum concentrations mean value of 17.2 pmol/L; sPH, severe primary hypothyroidism; TSH, thyrotropin (thyroid-stimulating hormone).

The surplus of the serum samples were kept at −20 °C until the TSH molecules were characterized with the methods described below during the period 2002 to 2012. The study was made according to the approval of the Ethics Committee of the Medical Faculty of Uppsala University (Ups 01-367, 2001-09-13, still valid) for use of surplus of serum samples in clinical chemistry.

The serum samples from the medical students were obtained as previously described [16]. Their donations of serum samples were accompanied with health declarations, signed by each student, and with informed consents according to the Declaration of Helsinki of Ethical principles for medical research and with the approval of the Ethics Committee.

### Immunoassay of Serum TSH

The concentrations of TSH in serum samples and in separated fractions after electrophoresis were measured using a time-resolved sandwich fluoroimmunoassay (Delfia, A042-201, PerkinElmer-Wallac Oy,Turku, Finland). The hTSH Ultra Delfia assay, is highly specific for the TSH polypeptide heterodimer. The method permitted room-temperature measurements of the hormone directly in the 0.075M veronal (Sigma-Aldrich Chemie Gmbh, Germany) buffer at pH 8.7 eluted from electrophoreses. The TSH values were expressed in mIU/L using the Third International TSH Standard, National Institute for Biological Standards and Control, Code 81/565, as a reference. The detection limit of the hormone in fractions from electrophoresis was about 100 attogram (100 × 10^−18^ g).

The serum samples had been kept deep-frozen for a period of 2 weeks up to 5 years when thawed for electrophoretic analyzes.

### Frequency of Glycoforms of TSH and AMS Residues per Glycoform Molecule

All serum samples were analyzed with an electrophoretic technique using an 0.10% agarose suspension in veronal buffer at pH 8.7 [9]. The area under the curve for eluted TSH was resolved into peaks at the positions for different numbers of AMS residues per molecule. The frequencies of the 2 glycoforms of TSH in the serum samples and the median numbers of AMS residues per glycoform molecule were calculated from the distribution by electrophoresis using the TSH algorithm as described [9].

### Neuraminidase Treatment

The terminal SA residues were removed from the TSH molecules in serum samples by neuraminidase treatment at pH 5.6 for 24 hours at 37 °C, leaving the SU as the only AMS remaining on the molecules, as described [9]. The immunological TSH activities measured before and after neuraminidase treatment of serum samples were not significantly different when measured with the Delfia immunoassay.

### Determination of SU and SA on Glycoforms

The number of SU residues and the percentage of SU out of the AMS were determined for each glycoform as described [9]. The ratio of percentage SU to the total AMS per molecule on low versus fully N-glycosylated glycoforms was used in the formula to calculate the number of SU and SA per glycoform molecule. The mean ratio of percentage SU on TSHdi versus percentage SU on TSHtri was 1.23 (SD, 0.031; range, 1.19-1.31; n = 21).

### Statistical Analyses

The values presented in Table 2 are the arithmetic means with the SD or, when the distributions are geometric, the geometric means with the SD factor. Statistical comparisons were made by using the nonparametric Mann-Whitney test. A difference with a *P* value < 0.05 was considered significant. The statistical analyses were calculated using GraphPad Prism 9 for Windows.

**Table 2.** Concentration, N-glycosylation, Sulfonation and Sialylation of TSH Molecules in Serum Samples of Patients with Severe Primary Hypothyroidism Compared With That of Age-Matched Euthyroid Individuals

Group Diagnosis Number of individuals SexAge, mean (range) years	A Hypothyroid 14 younger women31 (20–42)	B Euthyroid 143 younger women26 (21–44)	A vs B	C Hypothyroid 12 older men & women69 (51–93)	D Euthyroid 16 older men & women64 (46–87)	C vs D	A vs C
			*P value*			*P value*	*P value*
Serum concentration, mIU/L							
	Geometric mean; SD factor	Geometric mean; SD factor		Geometric mean; SD factor	Geometric mean; SD factor		
TSHdi, conc.	155; 1.70	0.98; 1.52	*a*	50.9; 2.14	0.92; 1.49	*a*	*b*
TSHtri, conc.	89.9; 1.87	0.95; 1.51	*a*	37.8; 2.17	0.88; 1.61	*a*	*c*
TSHdi/TSHtri	1.72; 1.26	1.03; 1.21	*a*	1.34; 1.20	1.04; 1.16	*b*	*c*
TSH total, conc.	246; 1.74	1.94; 1.50	*a*	89.1; 2.14	1.81; 1.54	*a*	*c*
**Percentage of low and fully N-glycosylated TSH.**							
	Mean; SD	Mean; SD		Mean; SD	Mean; SD		
Percentage of TSHdi	63.2; 5.38	50.8; 4.63	*a*	57.3; 4.43	51.0; 3.90	*b*	*c*
Percentage of TSHtrie	36.8; 5.38	49.2; 4.63	*a*	42.7; 4.43	49.0; 3.90	*b*	*c*
Degrees of sialylation and sulfonation. Number of SA and SU residues per molecule and of AMS per glycan							
	Mean; SD	Mean; SD		Mean; SD	Mean; SD		
SU residues on TSHdi	2.13; 0.18	2.65; 0.14	*a*	2.22; 0.18	2.71; 0.15	*a*	*NS*
SU residues on TSHtri	2.44; 0.18	2.90; 0.12	*a*	2.51; 0.19	2.94; 0.13	*a*	*NS*
SA residues on TSHdi	1.44; 0.15	1.15; 0.13	*a*	1.43; 0.18	1.10; 0.15	*a*	*NS*
SA resudies on TSHtri	2.75; 0.19	2.39; 0.17	*a*	2.74; 0.21	2.32; 0.19	*a*	*NS*
AMS/glycan on TSHdi	1.78; 0.04	1.90; 0.03	*a*	1.83; 0.03	1.90; 0.02	*a*	*d*
AMS/glycan on TSHtri	1.73; 0.04	1.76; 0.03	*a*	1.75; 0.05	1.75: 0.06	*NS*	*d*

Statistical comparisons with nonparametric Mann Whitney tests.

Abbreviations: AMS, anionic monosaccharide; NS, not significant (*P* > 0.05); SA, sialic acid; SU, sulfonated N-acetylgalactosamine; TSH, thyrotropin (thyroid-stimulating hormone); TSHdi, low-N-glycosylated TSH with 2 N-glycans; TSHtri, fully N-glycosylated TSH with 3 N- glycans.

^*a*^ *P *< 0.0001.

^*b*^ *P *< 0.001.

^*c*^ *P* < 0.01.

^*d*^ *P *< 0.05.

^*e*^Degree of N-glycosylation expressed as percentage of fully N-glycosylated glycoforms, percentage TSHtri.

## Results

### Severe Primary Hypothyroidism

The N-glycosylation, sulfonation, and sialylation and the number of AMS per glycan of TSH molecules in serum samples of patients with severe primary hypothyroidism were compared with those of euthyroid individuals of the same sex and similar age. The mean values for younger (<45 years) women from estimations of 11 different glycosylation related variables, and the *P* values from statistical comparisons, are presented in Table 2. The groups of older (>45 years) women and of older (>45 years) men had statistically similar (*P > *0.05) mean values for all the 11 variables. As this was found for both the euthyroid and the hypothyroid individuals, the groups of the older men and women are presented as combined groups in Table 2.

#### N-Glycosylation of TSH

The concentrations of both TSHdi and TSHtri were significantly higher in the 2 hypothyroid groups compared with the corresponding groups of euthyroid individuals. The TSHdi and TSHtri concentrations in the younger women with severe primary hypothyroidism were significantly higher than the corresponding values for the group of older adults.

The degree of N-glycosylation of TSH, expressed as percent fully glycosylated TSH (TSHtri), was significantly lower in the hypothyroid than in the euthyroid individuals. The hypothyroid younger women had a significantly lower degree of N-glycosylation of TSH than the hypothyroid older adults.

#### Terminal SU and SA residues per TSH glycoform molecule

The sulfonation of TSH, expressed as number of SU residues per TSHdi and TSHtri molecule, was significantly decreased for both groups of hypothyroid patients compared with the euthyroid individuals. The sialylation, expressed as number of SA residues per TSHdi and TSHtri molecule, was significantly increased in the patients suffering from severe hypothyroidism. The mean ratios of the number of SU versus SA residues per TSHdi and TSHtri molecule were significantly decreased in the hypothyroid patients.

#### AMS per TSH glycoform glycan

The number of anionic monosaccharides (AMS), that is, the sum of the numbers of SU and SA residues, per glycan on TSHdi and TSHtri was significantly decreased in the younger hypothyroid women compared with the young euthyroid women. The group of older hypothyroid patients had significantly lower values for number of AMS on TSHdi compared with the euthyroid individuals, while the corresponding values for TSHtri were not significantly different. The younger hypothyroid women had lower number of AMS per glycan on both TSHdi and TSHtri than the group of older hypothyroid men and women.

### Levothyroxine Treatment

**Figure 2.**
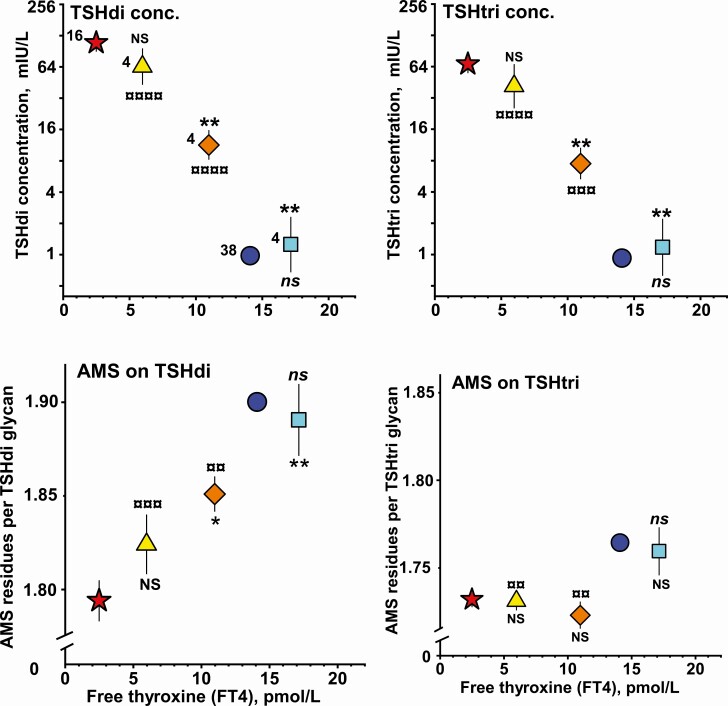
Relationship between mean levels of free thyroxine in serum versus TSHdi concentration (upper left panel), TSHtri concentration (upper right panel), anionic monosaccharide (AMS) residues per TSHdi glycan (lower left panel) and AMS residues per TSHtri glycan (lower right panel) for 3 groups of 4 women treated with levothyroxine for severe hypothyroidism (LevoT4-A, triangle; LevoT4-B, rhomb; LevoT4-C, square). The corresponding mean levels for 16 women with nontreated severe hypothyroidism (filled star) and 38 healthy women (filled circle) are shown. The vertical lines indicate ± SEM. NS, not significant; *, *P* < 0.05; **, *P* < 0.01, versus nontreated hypothyroid women. ns, not significant; ¤¤, *P* < 0.01; ¤¤¤, *P* < 0.001; ¤¤¤¤, *P* < 0.0001, versus healthy women.

**Figure 3.**
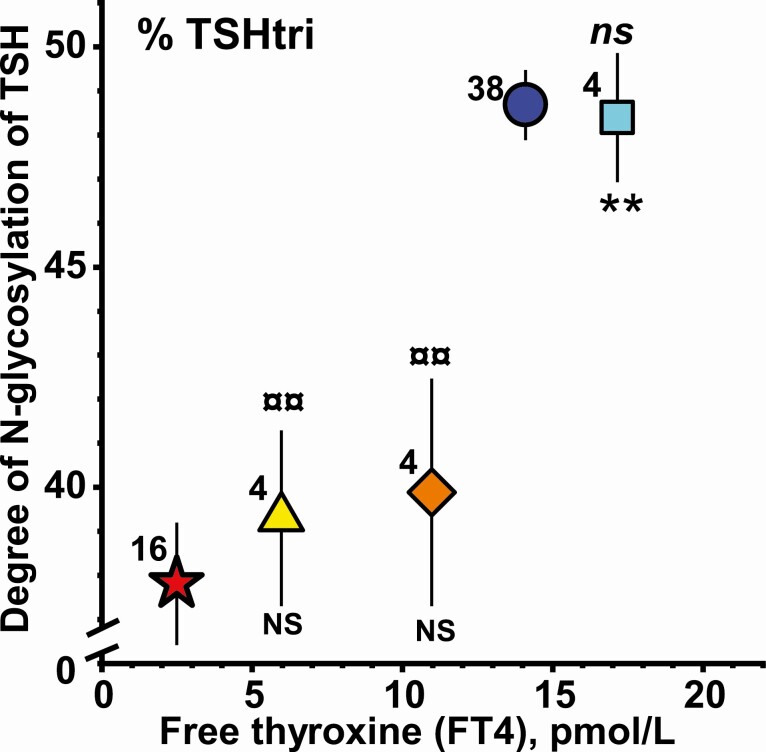
Relationship between mean levels of free thyroxine in serum versus degree of N-glycosylation of TSH for 3 groups of 4 women treated with levothyroxine for severe hypothyroidism (LevoT4-A, triangle; LevoT4-B, rhomb; LevoT4-C, square). The corresponding mean levels for 16 women with nontreated severe hypothyroidism (filled star) and 38 healthy women (filled circle) are shown. The vertical lines indicate ± SEM. NS, not significant; **, *P* < 0.01, versus untreated hypothyroid women. ns, not significant; ¤¤, *P* < 0.01, versus healthy women.

**Figure 4.**
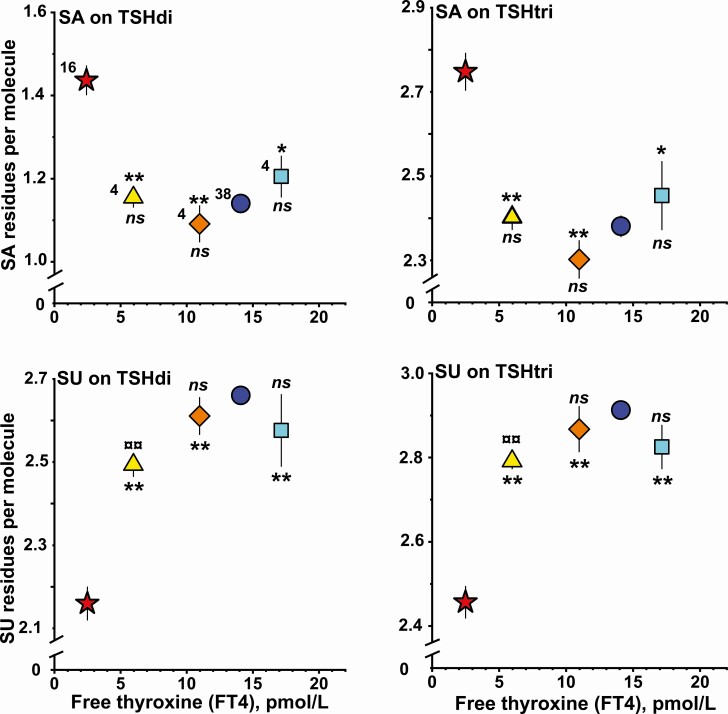
Relationship between mean levels of free thyroxine in serum versus number of SA residues per molecule on TSHdi (upper left panel), on TSHtri (upper right panel), number of SU residues per molecule on TSHdi (lower left panel), on TSHtri (lower right panel) for 3 groups of 4 women treated with levothyroxine for severe hypothyroidism (LevoT4-A, triangle; LevoT4-B, rhomb; LevoT4-C, square). The corresponding mean levels for 16 women with untreated severe hypothyroidism (filled star) and 38 healthy women (filled circle) are shown. The vertical lines indicate ± SEM. *, *P* < 0.05; **, *P* < 0.01, versus untreated hypothyroid women. ns, not significant; ¤¤, *P* < 0.01, versus healthy women.

The effects of the levothyroxine treatment on the glycobiological properties of the TSH molecules are presented in 9 diagrams (Figs. 2-4). The mean values ± standard error of the mean of 9 different variables are plotted in relation to the mean FT4 concentration. The statistical significance of difference for each of the 3 levothyroxine-treated groups of women versus the group of women with untreated severe primary hypothyroidism and the group of euthyroid women are indicated in the 9 diagrams.

#### TSH glycoform concentration

The serum concentrations of TSHdi and TSHtri versus those of FT4 are shown in Fig. 2, *upper panels.* The mean TSH concentration, both in the LevoT4-B group and in the LevoT4-C group, was significantly different from that of the group of women with untreated severe primary hypothyroidism. The mean TSH concentrations, both in the LevoT4-A group and in the LevoT4-B group, were significantly different from those of the euthyroid group. There was a significant inverse correlation of the 28 serum concentrations of FT4 of the 4 hypothyroid groups, the untreated and the 3 levothyroxine-treated, versus the log values of TSHdi or TSHtri (*r* = −0.89 and −0.87; n = 28; both with *P < *0.0001).

#### AMS residues per TSHdi glycan

The number of AMS residues per glycan on TSHdi and TSHtri are shown in Fig. 2, *lower panels.* The mean number of AMS residues per glycan on TSHdi, both of the LevoT4-B group and of the LevoT4-C group, was significantly different from that of the group of women with untreated severe primary hypothyroidism. The mean number of AMS residues per glycan on TSHdi, both of the LevoT4-A group and of the LevoT4-B group, was significantly different from that of the euthyroid group. There was a significant correlation between the FT4 concentration values of the 4 hypothyroid groups and the number of AMS residues on TSHdi (*r* = 0.74; n = 28; *P < *0.0001). There was also a significant inverse correlation between the log TSHdi concentration and the number of AMS per TSHdi molecule (*r* = −0.66; *P < *0.0001).

#### AMS residues per TSHtri glycan

The number of AMS residues on TSHtri of the 3 levothyroxine-treated groups were not significantly different from that of the group of women with untreated severe primary hypothyroidism. The LevoT4-A and LevoT4-B groups had significantly lower number of AMS residues than the group of euthyroid women. The correlation between FT4 values and number of AMS residues on TSHtri of the 4 hypothyroid groups (n = 28) was not significant. The mean values for AMS on TSHdi of all 5 groups were higher than the corresponding values on TSHtri.

#### Degrees of N-glycosylation

The relationships between the degrees of N-glycosylation of TSH, expressed as percentage TSHtri and the FT4 concentrations, are shown in Fig. 3. During the levothyroxine treatment the N-glycosylation is about 40% for both the LevoT4-A and LevoT4-B groups and not significantly different from that of the group of women with untreated severe primary hypothyroidism which occurred when the FT4 values reached a level of 14 to 22 pmol/L (LT4-C group).

The degrees of N-glycosylation of TSH of the groups LevoT4-A and LevoT4-B were significantly lower than that of the euthyroid group.

#### SA and SU residues per TSH glycoform molecule

The number of terminal SA and SU residues per molecule on TSHdi and TSHtri for the 5 groups of individuals are shown in the 4 panels of Fig. 4. In all levothyroxine treatment groups, the number of SA residues is significantly lower and the number of SU residues significantly higher compared with the group of women with the untreated severe primary hypothyroidism. The mean value of SU residues on TSH of the group LevoT4-A, with FT4 values around 6 pmol/L, was significantly different when compared both with the group of euthyroid women and with the group of women with untreated severe primary hypothyroidism.

## Discussion

The present study is the first report on the serum levels and properties of the different glycoforms of TSH in patients with severe primary hypothyroidism. The TSH molecule is a heterodimer of 2 polypeptides consisting of an alpha-subunit common to TSH, FSH, LH, and chorionic gonadotropin, and a noncovalently linked beta-subunit, which confers hormone specificity. The alpha-subunit is produced in large excess compared with the beta-subunit, ensuring that enough of diglycosylated alpha-subunits are synthesized.

TSHdi and TSHtri are secreted as spectra of very large numbers of forms differing in glycan structure determined by the actual number of terminal SA and SU residues per molecule. Our studies indicate that all these different TSH isoforms are present in serum of every individual. The relative abundance of the 2 glycoforms and of the more or less sialylated and sulfonated glycoforms in circulation vary both within and between individuals and are related to different clinical and physiological situations. The different forms are cleared from the circulation according to their individual clearance rate.

The composition of the isoform spectra of the 2 TSH glycoforms analyzed in a serum sample represents the isoforms circulating at the moment when the blood sample was taken. In the physiological and clinical studies, we assume that this composition is close to that reaching the target cells, in this case the TSH receptors on both thyroidal and extrathyroidal tissues, as for example, fat and bone tissues [17]. The biological effect of such isoform spectra will be a resultant of that of all the multiple isoforms.

Therefore, in our physiological and clinical studies, we have chosen to express, for each serum sample, the number of AMS, SU, and SA per glycoform molecule or per glycan as the median value of that of the multiple isoforms [9].

We report in this study that in patients with severe primary hypothyroidism, the majority of circulating TSH molecules differ from those in euthyroid individuals by being more sialylated and less sulfonated.

Both SU and SA regulate biological properties of TSH. TSH molecules with 2 or more SU residues are quickly cleared from blood circulation due to a SO_3_-N-acetylgalactosamine-receptor (SU-receptor) in the liver [18, 19]. The high degree of sialylation of the TSH molecules in patients having a severe primary hypothyroidism is in agreement with previous reports [2, 3, 5] and favors a prolonged survival in the circulation and a low biopotency of the TSH molecules at the target cells [7, 8]. Such a decreased biopotency may be essential to limit the effects of the high concentrations of circulating TSH on tissues with extrathyroidal TSH receptors [17].

The observation that serum TSH concentration in the group of younger women with severe primary hypothyroidism was higher than in the groups of older women and men is in agreement with previous reports [20-22]. We have previously reported that in euthyroid individuals, the amount of TSH extractable from individual pituitary glands of 7 older women was only 40 % of that of 12 young women [23]. This indicates an age-related decrease of the number of thyrotrophs in the pituitary gland or a decreased capacity of these cells to synthesize the TSH molecules in older individuals.

Patients with severe primary hypothyroidism demonstrate, when compared with euthyroid individuals, highly raised serum concentrations of TSH, low degrees of N-glycosylation, increased number of SA residues and decreased number of SU residues per TSH glycoform molecule and low number of AMS residues on TSHdi. When patients with severe primary hypothyroidism were treated with levothyroxine and received FT4 levels within the normal range, all these biochemical deviations were restored to normal. Several studies have shown that the FT4 serum concentration works as an excellent marker reflecting the clinical severity of primary hypothyroidism [24, 25]. The results of the present study show that the normalization of the biochemical deviations observed on circulating TSH molecules occurred at different levels of FT4 serum concentration.

The first variable to be restored was the synthesis of SA and SU residues on the glycans, occurring in the Golgi apparatus, and this eventuated for SA at a FT4 concentration as low as 6 pmol/L. The TSHdi and TSHtri concentrations that depend on production and secretion rates of the hormones, were restored gradually with the increased FT4 serum concentrations. The number of AMS on the TSHdi molecules increased gradually with increased FT4 concentration. Finally, the degree of N-glycosylation, occurring co-translationally in the endoplasmic reticulum of the thyrotrophs in the pituitary, remained at the low “hypothyroid level” of about 40% even when the FT4 levels had increased to the range of 11 to 12 pmol/L. This variable did not reach the euthyroid level of about 50% until the FT4 level was 17 pmol/L.

It is well established that there is a negative feedback relationship between the circulating thyroid hormones levels and the synthesis and secretion of pituitary TSH [8, 26]. In the present study, the inverse correlation between the concentration of FT4 and the log concentration of each of the 2 TSH glycoforms was very high (*P* < 0.0001). The number of AMS per TSHdi molecule increased in parallel with the FT4 concentration. This increase in AMS per TSHdi molecule is most likely an effect of the decreased TSH synthesis during the levothyroxine treatment. The capacity to add the terminal AMS groups in the Golgi on TSHdi may be limited when the TSH synthesis is increased. The capacity to add AMS per glycan on the TSHtri molecules, having 1 more glycan than TSHdi, did not change during the levothyroxine treatment.

Persani et al [7] reported a significant inverse correlation for the ratio of in vitro biological to immunological activity of TSH versus the degree of sialylation. In the patients with nontreated severe primary hypothyroidism, the 2 circulating TSH glycoforms had high contents of sialic acid per molecule indicating that the TSH molecules have a low biological potency at the target cells [7, 8]. During treatment with levothyroxine, the number of SA residues per TSH molecule decreased already at the FT4 serum concentration of 6 pmol/L and then remained low when the FT4 serum levels were raised to normal, favoring a higher biopotency of the TSH molecules.

Changes in the estimated degree of N-glycosylation, here expressed as %TSHtri in the serum samples, may be due to changes in the pituitary secretion of the 2 TSH N-glycoforms and/or to changes in elimination rates from the circulation for TSHdi and TSHtri glycoforms [10]. The biochemical events leading to this very early restoration to euthyroid levels of number of SA and SU residues during the levothyroxine treatment and, in contrast, the very late restoration of the N-glycosylation of the TSH molecules have not been elucidated. Persani [27] suggested that the hypothalamic thyrotropin-releasing hormone could play a major role modulating the biosynthetic processes within the thyrotrophs of the pituitary gland. This remains to be explored.

We observed that different biochemical deviations were restored at particular FT4 serum levels during the levothyroxine treatment of patients with severe primary hypothyroidism. However, this does not necessarily mean that the same FT4 serum levels are decisive when FT4 levels gradually decrease during progression from milder to severe primary hypothyroidism.

In conclusion, in patients with severe primary hypothyroidism, the serum concentrations of the 2 TSH glycoforms were extremely high. The TSH molecules were less N-glycosylated, resulting in an increased percentage of TSHdi compared with euthyroid individuals. Both the TSHdi and TSHtri molecules were more sialylated, less sulfonated, and had a lower number of AMS per glycan than in euthyroid individuals. This glycan pattern with both a high degree of sialylation and a low degree of sulfonation favors a prolonged half-life in the circulation. The high degree of sialylation favors a low biopotency at the target cells that may be essential to limit the TSH effects on tissues with extrathyroidal TSH receptors. All the biochemical deviations investigated in the group of patients with untreated severe primary hypothyroidism were restored to euthyroid levels by adequate treatment with levothyroxine. During the levothyroxine treatment of these patients the glycan decorations with SA and SU residues were the first to be restored to euthyroid levels, already at low FT4 serum concentrations. The decrease of the TSH serum concentration occurred gradually and was inversely correlated to the increased FT4 levels. The restoration of N-glycosylation of TSH did not occur until the FT4 serum concentration had reached euthyroid levels.

## Data Availability

Some or all data generated or analyzed during this study are included in this published article or in the data repositories listed in References.

## Abbreviations

AMS: anionic monosaccharide
FT4: free thyroxine
FSH: follicle-stimulating hormone
LH: luteinizing hormone
SA: sialic acid
sPH: severe primary hypothyroidism
SU: sulfonated N-acetylgalactosamine
TSH: thyrotropin (thyroid-stimulating hormone)
TSHdi: TSH with 2 N-glycans
TSHtri: TSH with 3 N-glycans

## Additional Information

***Disclosures:*** The authors have nothing to disclose.
